# Identification of the individual cardiac contraction threshold during high‐frame‐rate stress echocardiography

**DOI:** 10.1113/EP093437

**Published:** 2026-02-26

**Authors:** Fabian Spahiu, Max Hagemann, Michelle Ottlik, Moritz Lampkemeyer, Eric J. Stöhr

**Affiliations:** ^1^ COR‐HELIX, Institute of Sport Science Leibniz University Hannover Hannover Germany

**Keywords:** cardiac contraction threshold, exercise echocardiography, left ventricular mechanics, myocardial strain, stroke volume plateau

## Abstract

The clinical assessment of cardiovascular function during exercise using stress echocardiography is essential for accurate cardiac diagnosis. However, normal limitations of cardiac deformation responses to increasing physical exertion remain poorly understood. We introduce the individual cardiac contraction threshold (iCarT) as a novel marker of mechanical cardiac limitation and examine its relationship to standard cardiovascular measures.High‐frame‐rate (121–276 frames/s) exercise echocardiography was performed during stepwise cardiopulmonary exercise testing on a recumbent cycle ergometer in 19 healthy, recreationally active adults. Visit 1 was used to individualie exercise protocols, applied in Visit 2. Left ventricular (LV) peak twist, longitudinal strain (LS), and apical circumferential strain (ACS) were assessed at each exercise stage. Polynomial curve fitting was used to identify plateaus in deformation parameters. iCarT was defined as the relative heart rate, expressed as a percentage of maximum heart rate (% HR_m__a__x_), at which the final deformation parameter reached its maximum. Associations between iCarT and the timing of plateau onset in volumetric and vascular measures were analysed using Spearman and Pearson correlations.At the group level (*n* = 19), LS plateaued first (76% HR_m__a__x_), followed by ACS (82% HR_m__a__x_) and LV twist (83% HR_m__a__x_). iCarT occurred at 89 ± 13% HR_m__a__x_. At the individual level, among participants with complete deformation data (*n* = 12), 50% did not show a clear deformation plateau. In participants displaying iCarT (*n* = 6), LV twist was the final parameter to level off in 83%. iCarT showed no association with stroke volume, cardiac output, end‐systolic volume, or ejection fraction. iCarT identifies individual limits of cardiac deformation during exercise, characterized by marked interindividual variability. Its independence from conventional cardiovascular measures highlights the added value of deformation‐based thresholds, with LV twist emerging as the most robust parameter during increasing effort.

## Abstract (highlights)

- **What is the central question of this study?**
  Is there an individual limit in cardiac contraction during exercise, and if so, does it correlate with other physiological parameters?
- **What is the main finding and its importance?**
  Using high‐frame‐rate stress echocardiography, this study introduces the iCarT and demonstrates that mechanical cardiac limitation occurs later during exercise than previously assumed and varies markedly between individuals. When present, left ventricular twist most frequently defines the mechanical limit. iCarT is independent of volumetric and vascular limitations, highlighting myocardial deformation as a distinct dimension of cardiac limitation during exercise.

## 1 INTRODUCTION

Stress echocardiography has become an integral part of cardiovascular imaging, offering dynamic, real‐time insights into myocardial function under physiological load. Originally established for the detection of myocardial ischaemia, its application has rapidly expanded to include assessments of contractile reserve, diastolic function, pulmonary pressures, and valvular behaviour. In recent years, the integration of speckle‐tracking echocardiography into stress protocols has further enhanced the diagnostic power of this modality by allowing quantification of myocardial deformation, including longitudinal strain (LS), circumferential strain (CS) and left ventricular (LV) twist. It is currently thought that these deformation parameters provide a more sensitive and nuanced view of myocardial function, and their behaviour under stress may reveal dysfunctions that are not detectable at rest or through standard volumetric or haemodynamic parameters alone (Smiseth et al., 2016, 2025).

The increasing clinical demand for early detection of subtle myocardial abnormalities has placed imaging‐based functional markers at the forefront of cardiac diagnostics. A retrospective study at Modena University Hospital showed that from 2004 to 2015, the annual number of stress echocardiograms rose continuously, accompanied by a marked shift from pharmacological to exercise‐based stress protocols (Barbieri et al., 2018). Deformation imaging during exercise is particularly relevant for populations in whom traditional diagnostic endpoints, for example wall motion abnormalities or ejection fraction (EF), remain inconclusive (e.g., inconclusive, including patients with normal EF but reduced exercise tolerance) or those with early‐stage cardiomyopathies (Jin et al., 2021; Kraigher‐Krainer et al., 2014). In such contexts, myocardial mechanics under physiological or pharmacological stress may uncover hidden impairments in contractile efficiency or reserve that precede overt structural remodelling (Voigt et al., 2003).

Interestingly, several studies have shown that deformation parameters do not always increase linearly with rising exercise intensity, even in healthy populations. Rather, a number of studies show that parameters of LV systolic deformation, such as LV twist and LS, may plateau or even decline at submaximal workloads, suggesting a physiological limitation of contraction mechanics before maximum workload is achieved (Stöhr et al., 2011; Doucende et al., 2010). This plateau typically occurs simultaneously with or after levelling off in stroke volume (SV) and may serve as an earlier and potentially more sensitive marker of cardiac limitations.

At present, however, even in healthy individuals it remains unclear whether: (1) different parameters of LV deformation peak at similar exercise intensities and (2) the peak in LV deformation is associated with the decline in SV or other haemodynamic parameters.

In this context, we propose the concept of an individual cardiac contraction threshold (iCarT), defined as a plateau or decline in myocardial deformation despite further increases in external workload and heart rate, thus indicating a fundamental change in the underlying cardiac physiology in relation to whole‐body demands. As such, iCarT fulfils the conceptual criteria of a physiological threshold and may represent a valuable diagnostic marker for identifying latent cardiac performance limitations in both clinical and preventive cardiology settings. However, the identification of iCarT remains methodologically challenging. The load‐dependence of strain and twist parameters, combined with the technical constraints of echocardiographic imaging under exercise conditions, can complicate their use in standardized diagnostic workflows. In particular, the accuracy of speckle‐tracking‐derived deformation values is highly sensitive to frame rate settings and image acquisition quality. Our previous work systematically examined the impact of echocardiographic frame rate on tracking accuracy and absolute deformation values of longitudinal and rotational deformation measures during incremental exercise. The findings underscore that frame rate is not merely a technical setting, but a critical determinant of valid measurements, especially during increased heart rates when myocardial motion becomes more rapid (Spahiu et al., 2025).

Beyond technical considerations, a second major challenge in the literature relates to the interpretation of averaged group data in general and especially in the field of exercise physiology (Chrzanowski‐Smith et al., 2020; Watanabe et al., 2014). Many studies report mean changes in cardiac mechanics across exercise stages, yet fail to account for substantial interindividual variability. This practice risks overlooking physiologically meaningful patterns, like the early onset of mechanical plateaus in some individuals, or the absence thereof in others. Such variability may reflect differences in training status, cardiac geometry, autonomic regulation, or even subclinical myocardial pathology and may be crucial for truly personalized cardiovascular diagnostics.

To address these gaps, the present study aimed to provide a comprehensive, integrative assessment of LV mechanical function and volumetrics during incremental aerobic exercise, extending beyond submaximal workloads to include maximal exertion to volitional fatigue, thereby capturing cardiac behaviour across the full spectrum of supine exercise intensities. Crucially, this would be the first study to report both group‐level and individual‐level data on myocardial mechanics under graded exercise, with standardized control of imaging parameters, including high‐frame‐rate echocardiography. We hypothesized that different LV systolic deformation parameters reach their maximal response at distinct, submaximal exercise intensities rather than plateauing simultaneously, and that the final deformation parameter to level off, defined as iCarT, coincides with the plateau in SV. Furthermore, we hypothesized that the exercise intensity at which iCarT occurs shows substantial interindividual variability that is not adequately captured by group‐averaged analyses.

## 2 METHODS

### 2.1 Ethical approval

The study was approved by the local ethics committee of Leibniz University Hannover, Germany (reference number EV LUH 14/2024). All procedures conformed to the principles outlined in the latest revision of the *Declaration of Helsinki*. Written informed consent was obtained from all participants prior to data collection.

### 2.2 Study design and population

A total of 21 healthy recreationally active individuals were recruited and tested. The datasets of three participants were excluded from the overall analysis due to insufficient echocardiographic image quality and/or technical issues with continuous blood pressure monitoring during Visit 2. As a result, the final analysis included data from 19 participants with a mean age of 26 ± 3.12 years, a mean height of 179.25 ± 8.09 cm, body mass of 76.8 ± 6.33 kg, a mean body mass index (BMI) of 23.82 ± 2.35 and a peak oxygen uptake (${\dot{V}}_{O_{2}peak}$) of 3362.31 ± 561.95 mL/min. Of the 19 participants, five (26%) were women. The mean resting brachial systolic blood pressure was 125 ± 11.28 mmHg, whereas the mean resting diastolic blood pressure was 77 ± 6.33 mmHg. The average pulse wave velocity (PWV) at rest was 5.16 ± 0.47 m/s. All baseline characteristics can be obtained from Table 1.

**TABLE 1:** Baseline characteristics and frame rate settings (*n* = 19).

Parameter	Mean ± SD
Age (years)	26 ± 3.12
HR (beats/min)	71 ± 11
RR.sys (mmHg)	125.47 ± 11.28
RR.dias. (mmHg)	77.32 ± 6.33
PWV (m/s)	5.16 ± 0.47
Height (cm)	179.25 ± 8.09
Body mass (kg)	76.83 ± 6.33
BMI (kg/m^2^)	23.82 ± 2.35
SV (mL)	72.58 ± 15.90
EF (%)	55.3 ± 4.2
*W*_p__e__a__k_ (W)	226.68 ± 39.16
HR_m__a__x_ (beats/min)	179 ± 12
Absolute ${\dot{V}}_{O_{2}\mathrm{peak}}$ (mL/min)	3362.31 ± 561.95
Relative ${\dot{V}}_{O_{2}\mathrm{peak}}$ (mL/min/kg)	42.02 ± 5.06
FPS – Ap4Ch	141.53 ± 11.88
FPS – PSA‐base	167.14 ± 24.27
FPS – PSA‐apex	233.00 ± 21.64

*Note*: Values are presented as means ± SD. Abbreviations: BMI, body mass index; EF, ejection fraction; FPS – Ap4Ch, frames per second used for apical 4 chamber sequences; FPS – PSA‐apex, frames per second used for parasternal short‐axis views at LV‐apex; FPS – PSA‐base, frames per second used for parasternal short‐axis views at LV‐base; HR, heart rate; HR_m__a__x_, maximal heart rate; PWV, pulse wave velocity; RR.dias., diastolic blood pressure; RR.sys, systolic blood pressure; SV, stroke volume; ${\dot{V}}_{O_{2}\mathrm{peak}}$, peak oxygen uptake; *W*_peak_., peak workload.

All participants underwent two consecutive laboratory visits as part of a standardized exercise testing protocol at the COR‐HELIX laboratory of Leibniz University in Hannover, Germany. Data acquisition took place between July 2024 and March 2025. Participants were recruited from lectures at the Institute of Sports Science of Leibniz University and via social media platforms. Inclusion criteria were an age of over 18 years and a physically active lifestyle, defined as engaging in exercise on more than 2 days per week. Exclusion criteria included any kind of known cardiac abnormalities and poor image quality during baseline echocardiography. The first visit (Visit 1) served as a screening to assess eligibility for study participation and to determine individual peak oxygen uptake (${\dot{V}}_{O_{2}\mathrm{peak}}$) and maximal work capacity (*W*_p__e__a__k_) in watts. In the second visit (Visit 2), all individuals underwent an individualized step‐wise exercise protocol in order to determine iCarT.

### 2.3 Exercise protocol and study design

Before exercise initiation of Visit 1, anthropometric data including height, body mass, blood pressure and PWV were collected from all participants. Blood pressure and PWV were measured using the oscillometric method with the Mobil‐O‐Graph (IEM, Aachen, Germany) after a 10‐min seated rest period. Brachial blood pressure was obtained in the seated position with the cuff at heart level. Three consecutive measurements were taken, separated by 2‐min intervals, and the values for both blood pressure and PWV were averaged for analysis. Height and body mass were assessed using a stadiometer and scale from Soehnle (Nassau an der Lahn, Germany). Subsequently, participants were placed in a supine position (backrest angle was fixed at 0° and was identical for all participants) on the recumbent bicycle ergometer (Ergoselect 1200, Ergoline, Blitz, Germany) to individually adjust the bike settings (e.g., seat distance/leg extension and pedal fixation) prior to the exercise test. Afterwards, a 5‐min resting phase was conducted to evaluate image quality and determine whether the participant was suitable for exercise echocardiographic assessment scheduled for Visit 2. If image quality was insufficient, the procedure was discontinued prior to the stress test, and the participant was excluded from the study. If the participant was classified as eligible, the stress test was initiated. The protocol at Visit 1 began at 50 W with 30 W increases every 3 min, until volitional exhaustion was reached. No left‐lateral tilt was applied during Visit 1. The obtained *W*_p__e__a__k_ was used to individualize the subsequent testing protocol for Visit 2.

At Visit 2, participants performed an individualized exercise protocol, starting at 40% of prior identified *W*_p__e__a__k_ with the workload increasing by 10% of *W*_p__e__a__k_ every 3 min. The test continued until 100% *W*_p__e__a__k_ or volitional fatigue was reached. Cardiac ultrasound imaging was performed during the final 90 s of each stage. Respiratory gases were continuously measured via breath‐by‐breath analysis (Quark, Cosmed, Schweinfurt, Germany). Continuous beat‐to‐beat blood pressure was recorded using photoplethysmography (FinometerPRO, Finapres Medical Systems, Enschede, Netherlands). Throughout testing, a dedicated investigator continuously monitored the Finometer signal, provided real‐time feedback to participants to minimize movement, and intervened when motion artefacts occurred to ensure stable signal quality and valid blood pressure recordings. Systemic vascular resistance (SVR) was calculated according to the standard formula (MAP × 80)/$\dot{Q}$, with MAP denoting mean arterial pressure (mmHg) and $\dot{Q}$ denoting cardiac output (L/min) (Klabunde). SVR was thus expressed in dyn s cm^−^^5^. Vascular conductance (VC) was calculated as cardiac output divided by mean arterial pressure (VC = $\dot{Q}$/MAP) (Fairfax et al., 2013). Participants exercised in a supine position and were tilted to the left during the 90 s of data acquisition to enable recording of the targeted ultrasound sequences. Left‐lateral positioning during image acquisition was standardized to 45° using the tilt mechanism of the ergometer. Tilting was restricted to the acquisition window to optimize acoustic windows while limiting prolonged posture‐related alterations in physiological responses and cycling mechanics. Importantly, echocardiographic outcomes (and thus deformation‐derived iCarT) were acquired consistently under the same acquisition posture at each stage, thereby standardizing any tilt‐related influence on the imaging measures across intensities. A consistent cadence of 60 to 70 revolutions per minute was maintained throughout the test.

### 2.4 Echocardiographic image acquisition

For the quantification of two‐dimensional speckle tracking data, ultrasound images were acquired using a commercially available ultrasound system (Vivid E95, GE Medical, Horton, Norway). All data were collected by a trained cardiac sonographer using a multifrequency transducer (M5Sc D XDclear matrix single crystal phased array, GE Medical Systems Israel Ltd, Tirat Hacarmel, Israel). Data acquisition followed the previously described protocol by Stöhr et al. (2016). For the purposes of this study, the apical four chamber view, the parasternal short‐axis view at the LV base and the parasternal short‐axis view at the apex of the LV were obtained at rest and during the final 90 s of each exercise stage.

The LV base was defined as the short‐axis view showing the mitral valve leaflets during early diastole. The LV apex was identified by aligning the transducer along the long axis of the LV, identifying the optimal four‐chamber position and then moving caudally as little as possible, in a short‐axis angle, aiming to visualize the true apex while ensuring a circular lumen throughout the cardiac cycle and avoiding lumen obliteration at end systole. Each of the abovementioned images was acquired by using the highest frame rate possible in order to ensure optimal tracking quality and accuracy (Spahiu et al., 2025). This was achieved by setting the frame rate to its highest possible value while minimizing the imaging sector in terms of depth and width, ensuring that all relevant anatomical structures required for the quantification of myocardial deformation remained visible and the frame rate could be maintained as high as possible. The frequency of the M5Sc‐D transducer was always set to 1.7/3.3 MHz. The frame rates used for echocardiographic image quantification are shown in Table 1.

### 2.5 Echocardiographic data analysis

Echocardiographic image sequences were analysed offline using EchoPAC software (version 112, GE Vingmed, Horten, Norway) for initial data extraction by following the established guidelines (Gorcsan & Tanaka, 2011). For the analysis of parasternal short‐axis views of the apex and LV base, the commercially available software EchoPAC version 204 (GE, Trondheim, Norway) was used. Within the program, the $\dot{Q}$ analysis mode was selected, followed by the two‐dimensional strain option. The region of interest was then manually tracked and adjusted if necessary. In order to quantify LV rotational mechanics and apical circumferential strain (ACS), the raw speckle‐tracking output obtained from the manufacturer's software was further processed by interpolating data to 600 points with equidistant time intervals during systole and diastole, a method previously recommended (Burns et al., 2008), by using a custom software tool (2D Strain Analysis Tool, version 1.0beta14, Stuttgart, Germany). Basal rotation (measured in degrees) and rotation velocity curves (measured in degrees per second) were subtracted from the apical curves, respectively, to calculate peak systolic LV twist (expressed in degrees). We focused on ACS strain because prior work indicates that apical mechanics largely determine global circumferential strain (Beaumont et al., 2018). This obviated the need for an additional mid‐ventricular slice, thereby reducing acquisition time and susceptibility to echocardiographic artifacts and speckle‐tracking errors.

LS was determined using the Automated Function Imaging (AFI) feature available in EchoPAC software (version 204, GE Ultrasound) based on the apical four chamber view. AFI applies artificial intelligence for fully automated tracking of myocardial speckles, with the option to manually adjust regions of interest to ensure accurate tracking. This combination of automated tracking and manual refinement allows for precise assessment of LS, enhancing the reliability and reproducibility of the results. In addition, AFI enables the estimation of volumetric parameters such as end diastolic volume (EDV), end systolic volume (ESV), EF, SV and cardiac output ($\dot{Q}$) based on endocardial border tracking within the same apical four chamber recordings.

Owing to inadequate apical four‐chamber images during at least one exercise stage, LS and volumetric measures could not be obtained in 2 of 19 participants; only rotational metrics were available for analysis. Conversely, in another four participants, rotational and circumferential data could not be obtained. As a result, LS and volumetric outcomes could be analysed in 17 participants, and rotational/circumferential data were likewise available in 15 participants. Full data sets, including longitudinal, circumferential and rotational data, were available in 12 participants.

### 2.6 Determination of the individual cardiac contraction threshold (iCarT) and volumetric and haemodynamic plateaus

For each participant as well as for the whole study cohort, curve fitting analyses were conducted to investigate the relationship between relative exercise intensity expressed as a percentage of maximum workload (% *W*_p__e__a__k_), relative heart rate expressed as a percentage of maximum heart rate (% HR_m__a__x_) reached during the test of Visit 2 and each myocardial deformation parameter (LS, LV twist and circumferential strain). Primary results are expressed in percentage of maximum heart rate (% HR_m__a__x_) as this provides a more direct and accurate representation of the heart's physiological stress during exercise. Unlike external workload, which can vary between individuals, heart rate provides a personalized metric of internal cardiac reserve. This focus on HR_m__a__x_ allows for a more precise analysis of the heart's intrinsic limits and adaptations, which was the main goal of the study. Polynomial regression (quadratic or cubic models) was applied to characterize the relationship between relative exercise intensity/heart rate and myocardial deformation parameters. The aim was to identify the point at which each parameter reached its maximum before showing a plateau or decline in the curve. The optimal regression model for each parameter was selected based on corrected *R* ^2^ values and visual inspection of the curve progression. For quadratic models, the maximum was determined using the vertex formula (𝑥 = −*b*/2*a*); for cubic models, the location of the maximum was derived by solving the first derivative and identifying the global maximum within the tested intensity range. The latest deformation parameter that reached its maximum was defined as the iCarT, as it reflects the point at which the myocardium reaches its functional contractile limit under exercise conditions. If no such functional limit could be identified, the value 1 was assigned. This value corresponded to each participant's maximum relative heart rate and indicated a linear response without any sign of levelling off. Conversely, a value of −1 was assigned in cases where a negative association was observed, such as a consistent decline across all stages. Importantly, the same analytical approach was applied for quantification of SV, $\dot{Q}$, end‐systolic volume and EF, as well as to blood pressure and other haemodynamic parameter limitations. In all cases, the timing of plateau or peak responses was determined relative to HR_m__a__x_ using identical curve fitting and threshold detection procedures, ensuring methodological consistency across myocardial mechanics, volumetric function and haemodynamic responses. All deformation measurements obtained across incremental exercise stages were treated as individual data points for plateau determination and group level relationships between cardiac deformation and volumetric parameters. The reported *n* therefore represents the total number of data points included for each parameter.

### 2.7 Statistical analysis

All statistical analyses were conducted using GraphPad Prism (version10.6.0; GraphPad Software, Boston, MA, USA) and IBM SPSS Statistics (version 30.0; IBM Corp., Armonk, NY, USA). Data are presented as means ± SD. Non‐linear regression models were applied to characterize the shape and behaviour of each cardiac deformation parameter in relation to relative heart rate and relative exercise intensity. For iCarT determination, individual and group level curve fitting approaches were used to capture subject‐specific and collective response patterns. Significance was set at an α‐level of 0.05. In order to assess whether iCarT was related to the individual levelling off of volumetric, blood pressure and peripheral haemodynamic parameters and investigate the relationship between different cardiovascular outcomes, Spearman or Pearson correlation was performed. The choice between Spearman and Pearson correlation was based on the distribution of the respective variables, as assessed by the Shapiro–Wilk test for normality.

## 3 RESULTS

### 3.1 LV deformation – iCarT

#### 3.1.1 Group‐level analysis of plateaus in cardiac deformation

**FIGURE 1:**
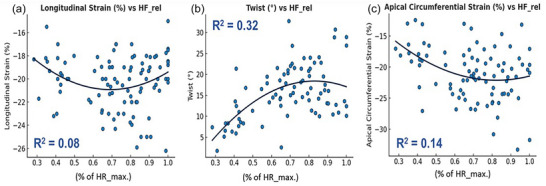
Scatter plots showing the relationship between relative heart rate (HR_m__a__x_) and cardiac deformation parameters during incremental exercise. Panels depict (a) longitudinal strain (*n* = 98), (b) LV twist (*n* = 82), and (c) apical circumferential strain (*n* = 86). Continuous lines represent fitted curves from polynomial regression. *R* ^2^ values indicate the proportion of variance explained by the model.

At group level, peak LV twist (*n* = 82), LS (*n* = 98) and ACS (*n* = 86) showed similar response patterns, characterized by an increase. All deformation parameters plateaued prior to *W*_peak_ and HR_m__a__x_, followed by a slight decline of varying magnitude between outcomes (Figure 1).

Curve fitting analysis at group level revealed a significant relationship between all deformation parameters and relative heart rate (% HR_m__a__x_; *P* < 0.001).

The timing, expressed as relative heart rate and relative exercise intensity, at which cardiac deformation parameters, volumetric outcomes and haemodynamic measures reached their maximum, is summarized in Table 2. LS reached its maximum first at 76% of HR_m__a__x_ (69% of *W*_peak_; *n* = 98), followed by ACS at 82% of HR_m__a__x_ (76% of *W*_peak_; *n* = 86), and peak LV twist at 83% of HR_m__a__x_ (86% of *W*_peak_; *n* = 82). iCarT was identified at 89 ± 13% of HR_m__a__x_ (*n* = 19).

**TABLE 2:** Group‐level curve fitting results (*n* = 19).

	% HR_m__a__x_		*W*_p__e__a__k_	
Parameter	Absolute	Percentage	Absolute	Percentage
iCarT	—	89	—	—
Twist (°)	18.32	83	18.8	86
LS (%)	−21.12	76	−21.1	69
ACS (%)	−22.14	82	−22.6	76
SV (mL)	86.15	68	85.8	40
EDV (mL)	137.63	60	138.8	34
ESV (mL)		−1		−1
EF (%)	63.34%	82	63	72
$\dot{Q}$ (L/min)	12.87	88	12.14	81
RR.sys. (mmHg)	185.49	77	180	73
RR.dias. (mmHg)		1		1
MAP (mmHg)	104.91	95	103.9	86
VC (mL/mmHg)	118.32	84	119	74
SVR (dyn s cm^−^^5^)	718.86	85	709.5	76

*Note*: Values represent the percentage of maximum heart rate (%HR_m__a__x_) or maximum exercise intensity (*W*_p__e__a__k_) at which the maximum of cardiac deformation blood pressure or haemodynamics could be detected. A value of –1 indicates a negative linear correlation and a value of 1 a positive linear correlation. Abbreviations: ACS, apical circumferential strain; EDV, end‐diastolic volume; EF, ejection fraction; ESV, end‐systolic volume; LS, longitudinal strain; MAP, mean arterial pressure; $\dot{Q}$, cardiac output; RR.dias., diastolic blood pressure; RR.sys, systolic blood pressure; SV, stroke volume; SVR, systemic vascular resistance; VC, vascular conductance.

**FIGURE 2:**
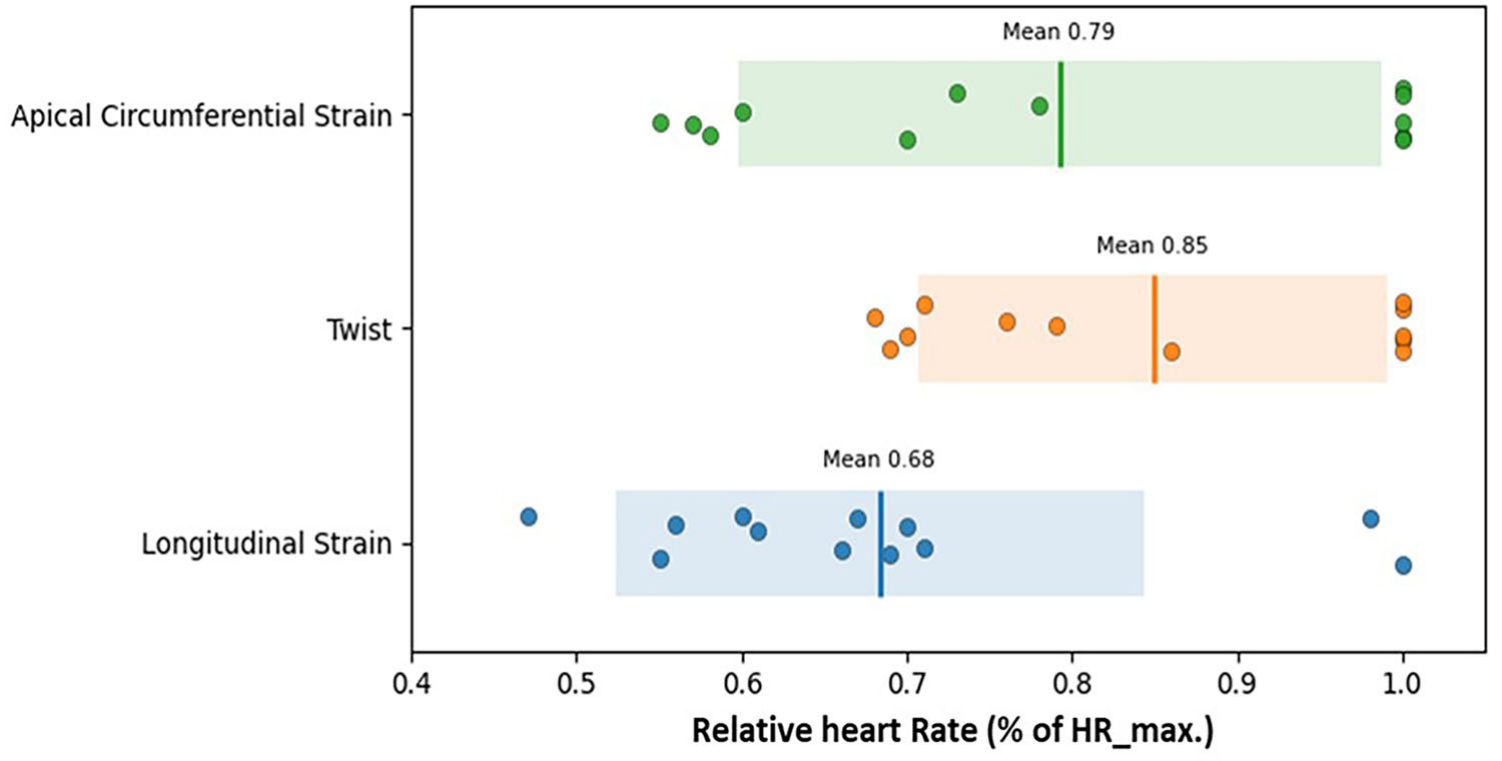
Individual onset of plateau in myocardial deformation parameters determined by curve fitting analysis. Individual relative heart rates (% of HR_m__a__x_) of the full data subgroup (*n* = 12) at which plateau responses occurred in myocardial deformation parameters: peak LV twist (orange), apical circumferential strain (ACS, green) and longitudinal strain (LS, blue).

In the subgroup with complete datasets (*n* = 12), mean values of the individually determined plateau points derived from curve‐fitting analyses showed an early onset in LS at 68 ± 15% of HR_m__a__x_, followed by ACS at 79 ± 19% of HR_m__a__x_, and a later onset in LV twist at 85 ± 14% of HR_m__a__x_. The mean onset values of these individual cardiac deformation plateaus are presented in Table 3. A graphical illustration of individual plateau points for the subgroup can be taken from Figure 2. In this subgroup, iCarT was detected at 90 ± 13% of HR_m__a__x_.

**TABLE 3:** Plateaus of LV deformation in full dataset and iCarT subgroup.

	Twist		LS		ACS	
	Mean	SD	Mean	SD	Mean	SD
Full datasets (*n* = 12)	0.85	0.14	0.68	0.15	0.79	0.19
iCarT subgroup (*n* = 6)	0.75	0.07	0.72	0.13	0.65	0.08

*Note*: Values are presented as means ± SD. The table summarizes curve fitting results for LV twist, longitudinal strain (LS), and apical circumferential strain (ACS) in all participants and in the iCarT subgroup.

#### 3.1.2 Individual – level analysis of plateaus in cardiac deformation

Individual‐level analysis revealed that, out of the total of 12 participants in which all deformation‐based data were available (full dataset), 50% (*n* = 6) showed a clear levelling off in cardiac deformation parameters, whereas in the other 50% of the participants (*n* = 6) no plateau could be detected. In 3 out of these 6 participants, peak LV twist and ACS increased throughout the entire exercise test. In one participant, solely LV twist showed no plateau, and in another participant, only ACS did not plateau during exercise. The subgroup analysis of those participants, who displayed iCarT (iCarT subgroup) showed that 83% (*n* = 5) exhibited the latest plateau onset in LV twist. Mean values of the individual plateau points in the iCarT subgroup, derived from curve‐fitting analyses, showed that ACS reached the earliest plateau at 65 ± 8% of HR_m__a__x_, followed by LS at 72 ± 13% of HR_m__a__x_, with LV twist showing the latest plateau at 75 ± 7% of HR_m__a__x_ (see Table 3). In this subgroup, iCarT was detected at 80 ± 11% of HR_m__a__x_.

### 3.2 Associations between relative heart rate and EDV, ESV, SV and $\dot{Q}$

At group level SV increased progressively with rising exercise intensity and reached its maximum at 68% of HR_m__a__x_. EDV showed an earlier levelling off, occurring at approximately 60% of HR_m__a__x_. In contrast, ESV exhibited a continuous, nearly linear decrease throughout the entire test. EF reached its maximum at 82% of HR_m__a__x_. $\dot{Q}$ also demonstrated a plateau, which occurred at 88% of HR_m__a__x_ (see Table 2). Mean and individual SV and $\dot{Q}$ responses for each participant are provided in the Supporting information.

Out of 17 participants, a plateau in SV was observed in 82% (*n* = 14), while a plateau in $\dot{Q}$ was identified in 65% (*n* = 11) of the total population. Regarding volumetric parameters, 52% (*n* = 9) showed a plateau in ESV, and 76% (*n* = 13) demonstrated a plateau in EDV. The exact individual data for volumetric measures and strain values can be found in the Supporting information.

### 3.3 Associations of relative heart rate with blood pressure and peripheral haemodynamics

Due to signal disturbances, blood pressure data from three participants had to be excluded, leaving valid data from 15 participants. Among these 15 individuals, cardiac output data were missing in two participants, resulting in SVR and VC being available for 13 participants. In all participants in which blood pressure data were available (*n* = 15), MAP reached its peak on average at 86% of HR_m__a__x_. VC and SVR reached their respective maximum values at 95% and 86% of HR_m__a__x_ (see Table 2). A consistent rise in systolic blood pressure was observed in 23% of participants (*n* = 3), in diastolic blood pressure in 54% (*n* = 7), in MAP in 46% (*n* = 6), in VC in 31% (*n* = 4), and in SVR in 31% (*n* = 4).

### 3.4 Relationships between cardiac deformation and volumetric parameter – group level and individual responses

**FIGURE 3:**
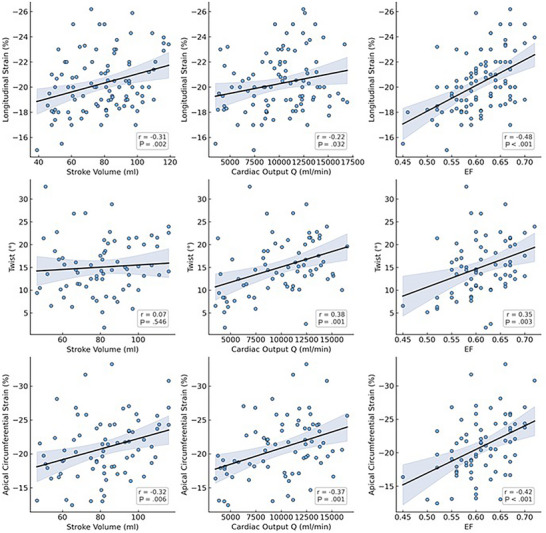
Multipanel scatterplots of myocardial deformation versus haemodynamic parameters. Rows show longitudinal strain (LS, *n* = 97), peak LV twist (Twist, *n* = 70) and apical circumferential strain (ACS, *n* = 70). Columns show associations with stroke volume (SV), cardiac output ($\dot{Q}$) and ejection fraction (EF). Each panel displays individual data points, an ordinary least‐squares fit in black, and a 95% confidence band in blue. Pearson's *r* and two‐sided *P* are reported within each panel.

At group level, significant positive associations between LS and SV (*r* = 0.31, *P* = 0.002), between LS and $\dot{Q}$ (*r* = 0.22, *P* = 0.048), and between LS and EF (*r* = 0.48, *P* < 0.001) were detected (*n* = 97). No significant association was observed between LV twist and SV (*P* = 0.546), while LV twist correlated significantly with $\dot{Q}$ (*r* = 0.38, *P* = 0.002) and with EF (*r* = 0.35, *P* = 0.004), based on 70 data points. ACS correlated significantly with SV (*r* = 0.32, *P* = 0.006), with $\dot{Q}$ (*r* = 0.37, *P* = 0.001), and with EF (*r* = 0.42, *P* < 0.001), also based on 70 data points. Overall, effect sizes were in the moderate range (see Figure 3).

At individual level, no significant correlations were detected between the occurrence of iCarT during stress echocardiography and the timing of plateau onset in SV (ρ = −0.31, *P* = 0.249, *n* = 16), ESV (ρ = −0.06, *P* = 0.829, *n* = 16), EDV (ρ = −0.04, *P* = 0.878, *n* = 17) and $\dot{Q}$ (ρ = −0.21, *P* = 0.427, *n* = 16). Moreover, no significant correlations between iCarT and the plateau onset of blood pressure measures like systolic (ρ = −0.04, *P* = 0.885, *n* = 15), diastolic (ρ = −0.15, *P* = 0.590, *n* = 15) and MAP (ρ = −0.09, *P* = 0.745, *n* = 15) could be found. No correlation between iCarT and plateaus of peripheral haemodynamic measures like SVR (ρ = 0.13, *P* = 0.679, *n* = 13) or VC (ρ = 0.20, *P* = 0.532, *n* = 13) could be detected.

In contrast, a significant positive correlation between the timing of levelling off in SV and $\dot{Q}$ (*r* = 0.58, *P* = 0.018, *n* = 16) could be observed. Moreover, a non‐significant correlation between the timing of plateau in LS and the timing of plateau and SV could be seen (*r* = 0.33, *P* = 0.211, *n* = 16).

## 4 DISCUSSION

This study identified the iCarT as a parameter that provides independent insight into myocardial performance during high‐frame‐rate stress echocardiography. The main findings of this study are that (1) LV twist seems to consistently plateau as the last deformation outcome in the majority of individuals – making it the most adequate deformation parameter in order to quantify cardiac contraction limitations; (2) the timing of iCarT occurrence shows large interindividual variability; and (3) iCarT does not correlate with plateaus in standard volumetric, blood pressure or haemodynamic measures.

### 4.1 The cascade of mechanical cardiac contraction limitations at group level

The most prominent finding was the considerable variability in the timing of plateau onset among LS, circumferential strain and LV twist at group level. While all these parameters increased progressively with exercise intensity/heart rate and plateaued before maximal work capacity/maximum heart rate, LV twist showed the latest plateau onset at ∼83% HR_m__a__x_ and ∼86% *W*_p__e__a__k_, highlighting its robustness as a marker of cardiac contraction limitations and iCarT. While LS describes the shortening of the LV long axis, it does not result from a dedicated layer of longitudinally oriented fibres. Instead, the helix‐like fibre arrangement throughout the myocardial wall generates circumferential and rotational components, with longitudinal shortening emerging as a net effect of these oblique fibre orientations and laminar sheet dynamics (Sengupta et al., 2008). Consequently, LS may plateau earlier, reflecting a more limited representation of global myocardial mechanics compared to ACS and LV twist. In contrast, LV twist arises from the counter‐directional rotation of apex and base and thereby integrates the coordinated contributions of subendocardial and subepicardial helices. This integrated mechanism provides greater functional reserve and may explain why LV twist continues to increase until higher workloads, highlighting its robustness as a marker of preserved contractile performance and its potential relevance for the definition of iCarT.

The observation that LS reaches its plateau earliest, followed by circumferential strain and finally by LV twist, is partly in line with the current body of evidence. Doucende et al. and Unnithan et al. reported that global LS plateaued early during endurance exercise in sedentary (30% of *W*_max_) and recreationally active individuals (40 W) (Doucende et al., 2010; Unnithan et al., 2015). While Doucende observed a continuous rise in circumferential strain and LV twist, Unnithan found an early plateau of circumferential deformation during upright endurance exercise, which occurred simultaneously with the LS plateau. In contrast, Pieles et al. (2021) showed in adolescent athletes an early flattening of LS accompanied by a continuous increase in circumferential strain in a semi upright CPET (seat position at 45°). Our findings are consistent with these patterns at group level but demonstrate a markedly later onset of plateau formation. Specifically, LS in our cohort plateaued at substantially higher workloads (∼156 W, 69% of *W*_max_), circumferential strain also showed a distinct plateau rather than a continuous rise, and LV twist – although the last parameter to plateau – clearly levelled off at ∼86% of peak power, later than previously described. Supporting this, Stöhr et al. (2011) demonstrated plateaus in LV twist, twist velocity, and untwisting velocity at ∼70% of peak power in recreationally active participants. Taken together, our results establish a cascade of plateau onset (longitudinal before circumferential and LV twist) but extend previous findings by demonstrating a systematically later plateau onset for all three parameters, including a distinct LV twist plateau not observed by Doucende et al. and occurring at higher relative intensity than reported by Stöhr et al. (Stöhr et al., 2011; Doucende et al., 2010). Differences between the mentioned findings and our results may be explained by study population characteristics as well as echocardiographic imaging protocols. One important methodological aspect concerns the frame rate used for image acquisition. Previous work from Fujikura et al. has shown that higher frames/s (FPS) improve temporal resolution and enhance accuracy in detecting end‐systolic and end‐diastolic frames (Fujikura et al., 2021). Doucende et al. applied a frame rate of 65–90 FPS, whereas Stöhr et al. used a consistent frame rate of 89 (data not reported in the original manuscript). Unnithan and colleagues stated that image‐acquisition settings were adjusted to maximize frame rate, although no specific numbers were reported, and Pieles and colleagues used frame rates ranging between 40 and 80 FPS (Doucende et al., 2010; Pieles et al., 2021; Stöhr et al., 2011; Unnithan et al., 2015). Given that the ESC guidelines recommend 40–80 FPS at rest with proportional increases during exercise (Voigt et al., 2015), it is plausible that lower frame rates were employed in most earlier studies compared to ours. The use of high frame rates (121–276 FPS in this study) likely enhanced tracking accuracy and may have contributed to the more precise detection of plateaus and delayed levelling off in cardiac mechanics in the presented study. Indeed, prior work from our lab has demonstrated that standard FPS settings (70–80) significantly underestimate absolute strain values at rest and particularly at high heart rates, potentially leading to misidentification of true myocardial thresholds (Spahiu et al., 2025). Consequently, high‐frame‐rates are essential for optimal tracking accuracy, and studies employing speckle‐tracking during exercise should explicitly report the FPS used to enable meaningful comparisons across studies.

### 4.2 Individual versus group‐level perspectives on cardiac deformation patterns

This study demonstrates that individual‐level analysis of cardiac contraction mechanics during CPET is essential for defining truly individualized physiological thresholds. In our subgroup with complete datasets, only 6 of 12 participants exhibited a clearly identifiable iCarT based on deformation parameters, emphasizing the limitations of relying exclusively on group‐level analyses to characterize cardiac mechanics. In contrast, six participants showed no discernible plateau in strain or LV deformation, indicating that mechanical limitation of cardiac contraction is not a universal feature of exercise physiology. When these findings are compared with the results from the preceding group‐level analysis, it becomes evident that population‐based averaging fails to capture the actual physiology of many individuals, as more than half of the participants did not demonstrate any cardiac limitation in one or more deformation parameters. These findings highlight the heterogeneity of myocardial responses to incremental exercise and underscore the need for individualized assessment rather than population‐level generalizations.

The cascade described above, in which LS plateaued first, followed by ACS and ultimately LV twist, was also confirmed by our individual‐level analysis. Among participants with complete datasets, most displayed this sequence of plateau onset, with LV twist consistently representing the final deformation parameter to reach a plateau (see Figure 2). This indicates that LV twist consistently plateaued later than longitudinal or circumferential deformation. Furthermore, in the subgroup without a discernible iCarT, it was most often LV twist and ACS that failed to demonstrate a plateau, whereas LS revealed a clear plateau in nearly all participants, with the exception of two cases (ID 011 and ID 008; data can be found in the Supporting information). An exception was the small subgroup of participants who displayed an iCarT, where the pattern suggested that ACS plateaued before LS, with LV twist still representing the final parameter to reach a plateau. Given the limited sample size in this subgroup (*n* = 5), this deviation must be interpreted with caution.

### 4.3 Coupling of myocardial deformation and volumetric output

At group level, our results in regard of the coupling of LS and SV seem to be in line with the results from Doucende et al., showing a significant relationship between LS and SV, thereby suggesting that SV is dependent on longitudinal shortening of the ventricle (Doucende et al., 2010). Moreover, a significant relationship between ACS and SV could be detected. In contrast to the observations of Stöhr et al. (2011), our data did not indicate a relationship between LV twist mechanics and SV, but rather between LV twist and $\dot{Q}$ as well as LV twist and EF (see Figure 3). A plausible mechanism is that torsional reserve provides partial compensation when rising heart rates shorten diastolic filling time, end‐diastolic volume approaches a plateau, and longitudinal shortening declines. By enhancing systolic ejection efficiency and supporting rapid diastolic suction through untwisting, LV twist mechanics help to sustain forward flow under these conditions. However, this compensatory mechanism does not prevent SV from plateauing or even decreasing at higher workloads, as observed in our data. Rather, it may limit the extent of SV decline and thereby help maintain overall cardiac output despite the exhaustion of LS. This interpretation aligns with the findings of Doucende et al., who observed a continued rise in LV twist even after LS had reached a plateau. These interpretations are correlational and should be validated with multivariable models that adjust for heart rate, loading conditions and cardiac geometry before broader generalization.

Nevertheless, group‐level data are insufficient to capture the true physiological relationship between cardiac contraction and volumetric measures at the individual level, since cardiac contraction limitations vary substantially between individuals, as outlined in the preceding section. Considerable interindividual variability also exists in regards of volumetric output, blood pressure and peripheral blood flow, which becomes more apparent when individual datasets are examined. Similar to cardiac contraction, interindividual variability has been noted for SV, $\dot{Q}$, MAP, VC and SVR responses during exercise, where not all individuals demonstrate a clear submaximal plateau (individual data can be found in the Supporting information).

**FIGURE 4:**
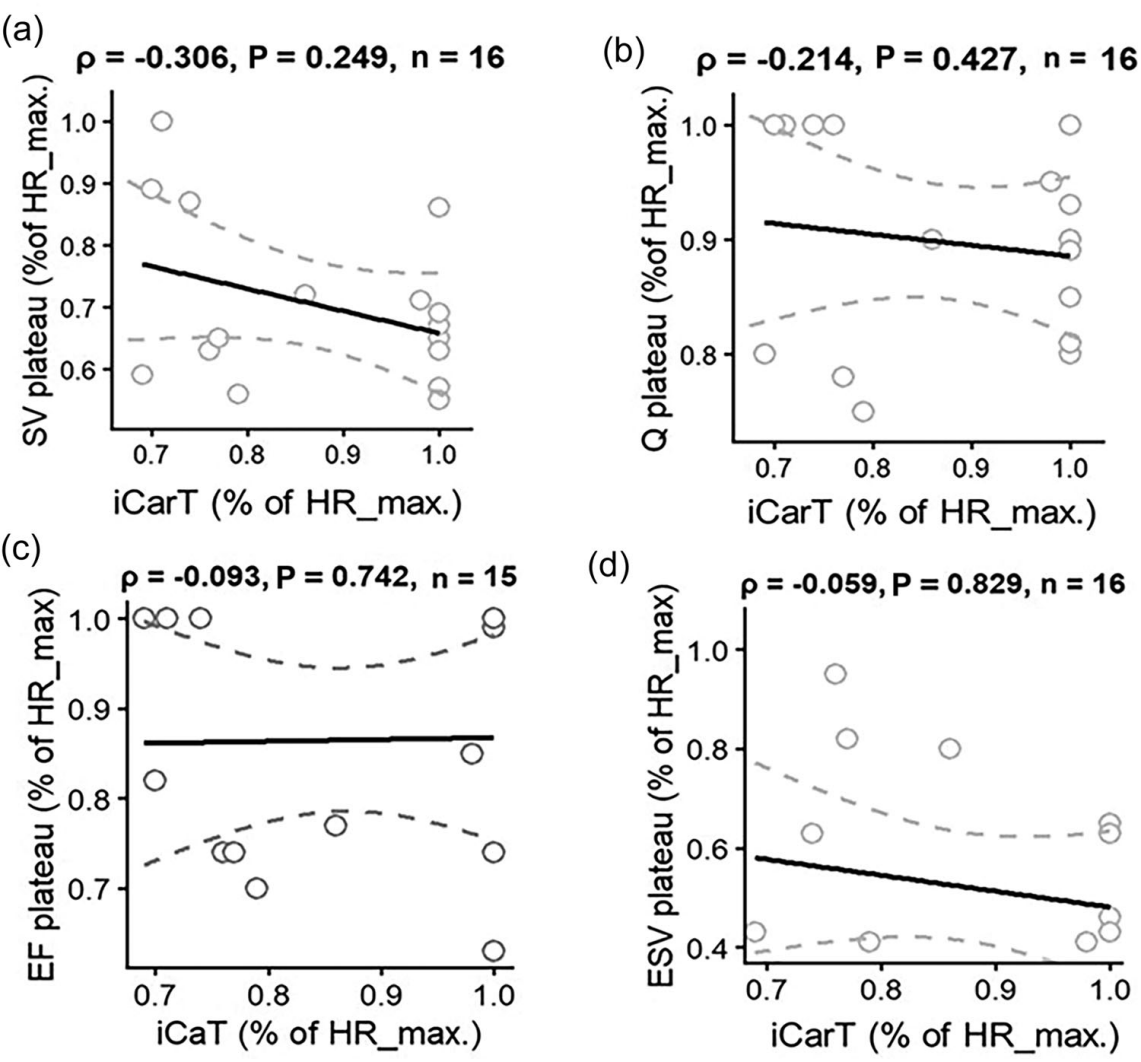
Associations between plateau responses of LS and SV and $\dot{Q}$ and SV. (a) Relationship between the plateau of longitudinal strain (LS) and the plateau of stroke volume (SV), expressed as percentage of maximal heart rate (%HR_m__a__x_). (b) Relationship between the plateau of SV and the plateau of cardiac output ($\dot{Q}$), expressed as %HR_m__a__x_.

**FIGURE 5:**
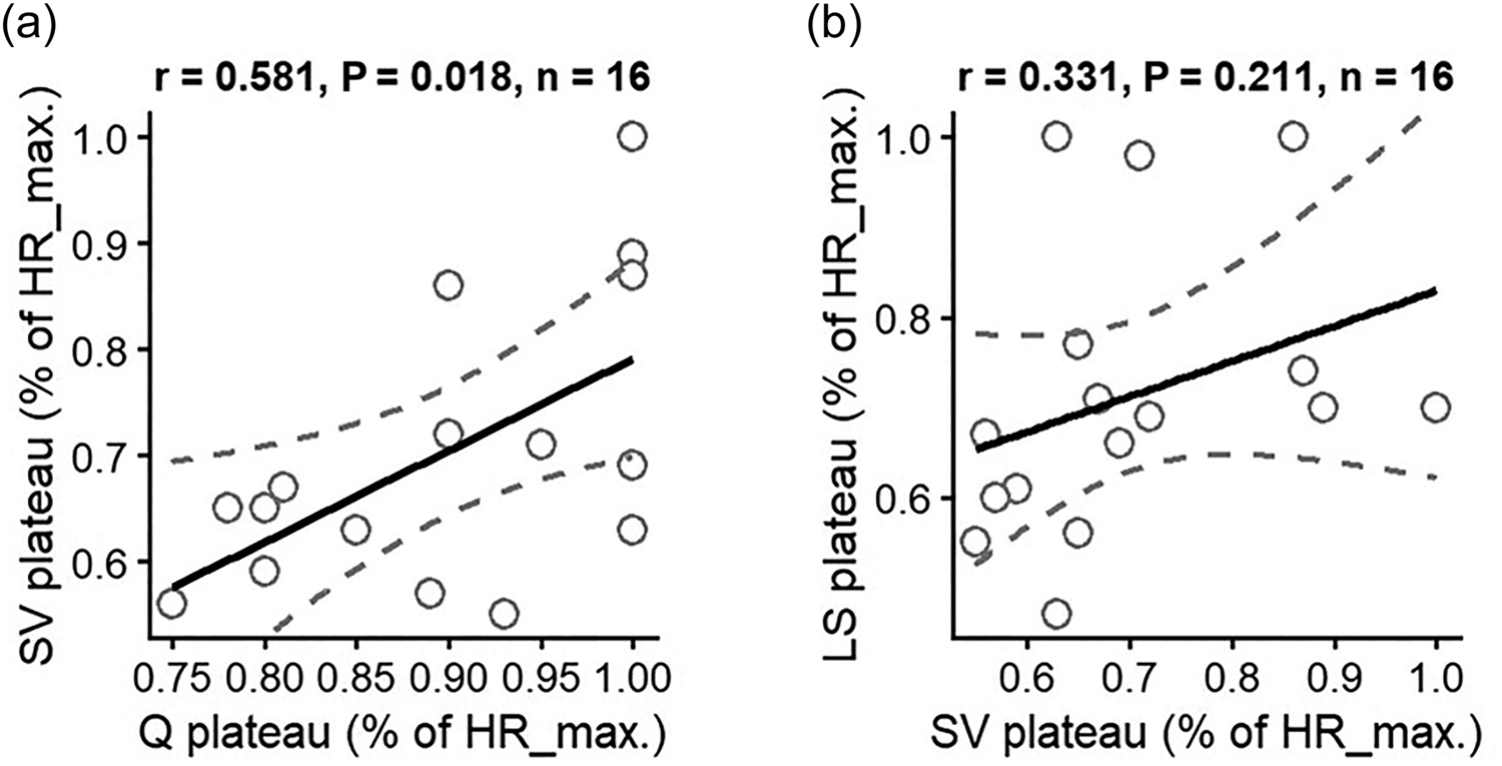
Relationship between the individual cardiac threshold (iCarT) and plateau levels of central cardiac parameters during incremental exercise. All parameters are expressed as percentage of maximum heart rate (% of HR_m__a__x_). (a) Stroke volume (SV) plateau in relation to iCarT (*n* = 17). (b) Cardiac output ($\dot{Q}$) plateau in relation to iCarT (*n* = 17). (c) End‐systolic volume (ESV) plateau in relation to iCarT (*n* = 17). (d) Ejection fraction (EF) plateau in relation to iCarT (*n* = 17). Each panel displays individual data points and corresponding regression lines. Reported *P*‐values indicate statistical significance, and *r* denotes the Pearson correlation coefficient.

Our individual‐level analyses demonstrated no significant linear association between the timing of the SV plateau and the onset of the LS plateau (Figure 4). This observation indicates that the plateau in SV cannot be attributed solely to myocardial deformation along the longitudinal axis. Moreover, no corresponding associations were identified between LV twist or circumferential strain and SV, $\dot{Q}$ or EF. Taken together, these findings suggest that while volumetric parameters are closely interrelated, the limitations observed in SV cannot be directly explained by mechanical constraints of myocardial deformation. It is conceivable that haemodynamics external to the heart, such as aortic blood pressure, play a greater role in the generation of SV during exercise. From a clinical perspective, this implies that cardiac mechanics during exercise appear to operate independently not only of volumetric measures such as SV and $\dot{Q}$ but also of systemic blood pressure, underscoring the need to consider additional mechanisms beyond central haemodynamics when assessing exercise‐induced cardiac limitations. Moreover, a dissociation between maximum myocardial mechanics, as defined by iCarT, and systemic haemodynamics, can be shown, since no significant correlations were observed between iCarT and the levelling off of volumetric measures, blood pressure or peripheral haemodynamics (data can be taken from Figure 5 and the Supporting information). This reinforces the view that global haemodynamic markers may fail to capture subtle impairments in myocardial contractile function, particularly during exercise when compensatory mechanisms obscure localized dysfunction. Deformation‐based thresholds such as iCarT may therefore provide a more sensitive tool for detecting early cardiac specific limitations, independent of standard volumetric measures. Consequently, iCarT, which in most participants was derived from LV twist, may serve as a sensitive marker for detecting myocardial impairment. Its application could be especially valuable in distinguishing early differences between healthy individuals and those with cardiac dysfunction during exercise. Supporting this notion, Peteiro et al. demonstrated that during exercise stress echocardiography, patients with preserved EF but inducible ischaemia showed significantly reduced LV twist and circumferential strain compared to non‐ischaemic controls (Peteiro et al., 2014). Moreover, LV twist during dobutamine stress has been identified as a strong predictor of LV remodelling after myocardial infarction (Joyce et al., 2014). These findings underscore the clinical relevance of LV twist mechanics – and potentially iCarT (as it is derived from LV twist in most individuals) – for detecting ischaemia‐related dysfunction and adaptive remodelling under stress conditions, independently of volumetric measures.

### 4.4 Limitations

First, our findings are specific to a recumbent exercise protocol with intermittent left‐lateral positioning during image acquisition. As shown by Beaumont et al., semi‐supine exercise and left‐lateral tilting can alter cardiorespiratory and haemodynamic responses compared with upright cycling, even at identical external workloads or matched heart rates (Beaumont et al., 2021). In particular, changes in body position affect venous return, preload, heart rate, SV, myocardial work (rate‐pressure product), oxygen uptake and mechanical efficiency (Beaumont et al., 2021; Wehrle et al., 2021).

We cannot fully exclude the possibility that posture‐dependent alterations in central and peripheral physiology may influence both the magnitude and the timing of iCarT. Consequently, the detectability and relative exercise intensity of the iCarT observed in the present study may differ in upright or continuously tilted stress‐echocardiography configurations, limiting direct generalizability across stress‐echo protocols. Nonetheless, the whole‐body coordination of all biological systems during supine, tilted exercise is most likely preserved, so that the interindividual comparisons are most likely relatively consistent. Furthermore, the fact that some individuals did not have an iCarT indicates that the present observations may be more related to individual biological differences rather than methodological approaches.

Second, in participants without an identifiable plateau, the classification of ‘no plateau’ may reflect termination of exercise before reaching the intensity domain at which a levelling‐off in myocardial deformation would have emerged. Repeated transitions into a left‐lateral position during Visit 2 may have increased internal load at a given external power output by altering heart rate–oxygen uptake relationships, as well as perceived effort, thereby promoting earlier volitional fatigue compared with a continuously non‐tilted protocol. To control for physiological exertion and ensure a high level of effort, a respiratory exchange ratio (RER) ≥ 1.10 was used as a criterion for maximal or near‐maximal exercise and was reached by all participants. However, RER‐based criteria have inherent limitations, as RER can be influenced by factors such as body position, ventilatory patterns, hyperventilation and non‐metabolic CO_2_ production, and therefore may not uniformly reflect comparable levels of peripheral or central fatigue across individuals or exercise configurations.

Third, our cohort comprised young, recreationally active adults. Accordingly, the present iCarT and plateau patterns should not be assumed to generalize to older individuals, highly trained endurance athletes, or clinical populations, in whom cardiac structure and function, autonomic control and exercise tolerance may differ. This might alter the propensity and timing of deformation plateaus.

### 4.5 Conclusion

Our results demonstrate that cardiovascular limitations during exercise are individualized and temporally asynchronous relative to external exercise intensity, rather than reflecting coordinated systemic events. iCarT represents a novel, non‐invasive marker of mechanical myocardial limitation that captures aspects of cardiac function not reflected by conventional volumetric or haemodynamic measures. The lack of association between iCarT and traditional cardiovascular indices suggests that deformation‐based thresholds provide complementary information on cardiac limitation during exercise. In this context, LV twist emerged as the most consistent marker of maximal myocardial deformation (iCarT) in the majority of participants, supporting its potential physiological and diagnostic relevance. Future studies should confirm the reproducibility and prognostic value of iCarT in larger and more diverse cohorts and further explore the interaction between central and peripheral plateaus to advance understanding of integrated cardiovascular responses to exercise stress. Ultimately, our results highlight that individualized analytic approaches are indispensable for unravelling the true physiology of cardiovascular responses to exercise and may represent the key to advancing both fundamental research and clinical diagnostics.

## AUTHOR CONTRIBUTIONS

The authors affirm that the results of the present study are presented clearly, honestly, and without fabrication, falsification, or inappropriate data manipulation. All experiments were performed in the COR‐HELIX laboratory at the Institute of Sports Science of Leibniz University Hannover. Fabian Spahiu and Eric J. Stöhr conceptualized and designed the study. Data acquisition was performed by Fabian Spahiu, Max Hageman, Michelle Ottlik, Moritz Lampkemeyer, and Eric J. Stöhr. Data analysis and interpretation were performed by Fabian Spahiu, Max Hagemann, and Eric J. Stöhr. Fabian Spahiu and Eric J. Stöhr drafted the manuscript. All authors critically revised the manuscript, approved its final version and agree to be accountable for all aspects of the work in ensuring that questions related to the accuracy or integrity of any part of the work are appropriately investigated and resolved. All persons designated as authors qualify for authorship, and all those who qualify for authorship are listed.

## CONFLICT OF INTEREST

The authors declare no conflicts of interest.

## FUNDING INFORMATION

This research received no external funding and was entirely self‐financed by the authors.

## Supporting information



Supplementary Figures S1–S3.

## Data Availability

The data that support the findings of this study are not publicly available due to ethical and data protection considerations. However, all raw data underlying the results presented in this manuscript will be made available by the corresponding author upon reasonable request, in accordance with the policies of *Experimental Physiology*.
