# Supplementary material for: Identification of the individual cardiac contraction threshold during high‐frame‐rate stress echocardiography
Source: Exp Physiol. 2026 Feb 26:10.1113/EP093437. Online ahead of print. doi: 10.1113/EP093437 (PMC13394920; doi:10.1113/EP093437)

Supplemental Files

Supplemental Figure 1. Individual plateau points of relative heart rate (%HR_max.) for Cardiac Deformation, Blood Pressure, Peripheral Hemodynamic and Volumetric Data

	LS		LV Twist		LV Untwisitng		LV Basal_Rot		LV Apical_Rot.		LV Basal_Circ.		LV Apical:Circ.		Ejection Fraction	
ID	HF_rel	Value_LS	HF_rel	Value_Twist	HF_rel	Value_Untw	HF_rel	Value_B.rot	HF_rel	Value_Ap.rot	HF_rel	Value_B.Circ	HF_rel	Value_Ap.Cir	HF_rel	Value_EF
ID_001	0,7	-19,84	0,71	15,49	0,72	-168,11	0,52	-1,45	0,69	12,61	0,67	-20,64	0,6	-21,77	1	0,68
ID_002	0,7	-24,65													0,82	0,62
ID_003	0,56	-18,66	1	19,3	0,62	-216,99	0,55	-8,87	0,53	7,59	0,69	-21,11	0,57	-27,08		
ID_004	0,67	-20,93	0,79	16,64	1	-147,13	0,67	-10,84	0,64	10,13	0,63	-15,93	0,55	-24,4	0,7	0,57
ID_005	0,47	-22,79	0,76	12,36	1	-255,98	0,72	-6,88	0,82	6,97	0,56	-21,97	1	-17,59	0,74	0,65
ID_006	0,74	-18,93					0,51	-2,62			1	-29,54			1	0,63
ID_007	0,61	-20,12	0,69	19,94	0,71	-246,61	0,77	-11,07	0,63	9,92	0,57	-11,67	0,58	-14,16	1	0,65
ID_008	1	-16,44													1	0,58
ID_009	0,77	-23,88													0,74	0,64
ID_011	0,98	-20,19	0,7	21	0,75	-281,45	0,6	-6,05	0,73	14,73	0,65	-17,64	0,7	-23,76	0,85	0,68
ID_012	1	-24	1	22,78	0,74	-240,68	0,52	-3,95	0,52	13,24	0,81	-10,4	1	-21,13	0,99	0,66
ID_014			1	24,1	1	-288,57	1	-10,2	0,83	13,3	-1	-17,26	0,79	-22,8		
ID_015	0,71	-23,3	1	28,89	0,79	-335,73	0,86	-7,26	1	24,65	0,83	-22,37	1	-33,24	0,74	0,7
ID_017			1	21,36	0,67	-303,89	0,52	-3,94	1	31,62	1	-23,53	0,63	-22,21		
ID_018	0,69	-19,09	0,86	14,09	1	-233,06	0,82	-5,05	0,88	10,86	0,69	-20,07	0,73	-23,53	0,77	0,63
ID_019	0,66	-20,82	0,68	32,96	0,73	-264,97	0,72	-10,59	0,67	20,94	0,93	-5,98	1	-29,76	1	0,65
ID_020	0,6	-21,62	1	30,72	0,94	-267,84	0,66	-6,4	1	34,78	0,12	-19,16	0,78	-23,72	0,63	0,64
ID_021	0,55	-21,69	1	13,84	1	-392,17	0,46	-2,07	1	4,18	0,66	-21,13	1	-25,78	1	0,67
Group Mean	0,71	-21,06	0,87	20,96	0,83	-260,23	0,66	-6,48	0,78	15,39	0,59	-18,56	0,78	-23,64	0,87	0,64
Group SD	0,15	2,19	0,14	6,21	0,14	60,25	0,15	3,18	0,17	8,90	0,47	5,62	0,18	4,52	0,13	0,03

	Stroke Volume		End Sys. Volume		End Dias. Volume		Cardiac Output		Sys. BP		Dias. BP		Mean Art. Pressure		Vasc. Conductance		Systemic Vasc. Res.		
ID	HF_rel	Value_SV	HF_rel	Value_ESV	HF_rel	Value_EDV	HF_rel	Value_Q	HF_rel	Value.RR.Sys	HF_rel	Value.RR.dia	HF_rel	Value_MAP	HF_rel	Value_VC	HF_rel	ValueSVR	iCarT
ID_001	1	105,42	-1	42	0,64	156,97	1	16924,8											0,71
ID_002	1	126,37	-1	62,5	1	154,68	1	16660	0,76	232,01	0,85	91,41	0,78	130,84	1	126	1	633,85	0,7
ID_003	0,65	67,4	0,65	67,4	1	131,88	0,8	8701,26	0,94	176,37	1	73,13	1	100	0,83	95,65	0,83	803,5	1
ID_004	0,56	102,12	-1	49	0,45	172,72	0,75	13017,65	0,71	180,41	0,72	73,75			0,67	153,42	0,68	523,12	0,79
ID_005	0,63	72	0,95	39,78	0,65	129,88	1	11045,51	0,86	155,12	0,99	62,29	1	89,55	0,92	121,78	0,87	653,19	0,76
ID_006	0,87	87,49	0,63	70,63	0,67	153,04	1	26365,52	0,65	158,71	1	61	0,52	75,26	1	181,79	1	278,94	0,74
ID_007	0,59	87,79	-1	37	0,53	142,14	0,8	11805,78	1	196					0,79	125,07	0,8	630,13	0,69
ID_008	0,63	58,15	0,63	58,15	0,64	105,88	0,85	8299,43	0,76	162,22	0,84	55,03	0,79	84,64	1	114,98	1	724,87	1
ID_009	0,65	85,38	0,82	45,06	0,25	136,58	0,78	10737,79	0,96	183,66	1	73,29	0,94	92,89	0,76	118,85	0,76	670,47	0,77
ID_011	0,71	99,37	-1	38	0,66	148,46	0,95	14505,37	0,84	234,15	1	103,18	0,93	136,15	0,92	107,1	0,9	742,22	0,98
ID_012	1	62,99	0,89	33,3	1	94,16	1		1	193	1	87	1	115	0,94	97,28	0,88	831,09	1
ID_014									0,72	190,83	1	102	1	127					1
ID_015	0,67	117,41	0,97	45,46	0,61	169,28	0,81	13339,79											1
ID_017									0,68	218,15	0,63	81,35	0,65	108,25					1
ID_018	0,72	100,06	0,6	60,3	0,69	160,4	0,9	16706,26	1	205,28	1	100	1	131	0,79	151,77	0,8	533,59	0,86
ID_019	0,69	49,87	-1	23,5	0,49	54,76	1	9897,07	0,74	193,56	0,64	103,28	0,69	132,66	1	74,26	1	674,19	1
ID_020	0,57	104,97	-1	50	0,49	167,98	0,89	13869,48											1
ID_021	0,55	92,94	-1	35	1	108,86	0,93	11802,16	0,93	173,62	0,86	79,1	0,88	103,71	0,93	114,02	0,87	695,41	1
Group Mean	0,72	88,73	-0,12	47,32	0,67	136,73	0,90	13578,52	0,84	190,21	0,90	81,84	0,86	109,77	0,89	121,69	0,88	645,74	0,89
Group SD	0,15	21,03	0,89	12,93	0,22	31,01	0,09	4330,45	0,12	23,60	0,14	15,87	0,15	19,79	0,10	26,89	0,10	136,24	0,13

Supplemental Figure 2. Relationship between iCarT and MAP, VC and SVR

Correlation Table ▼

Variable		iCarT	MAP
1. iCarT	Pearson's r	—	
	p-value	—	
	Spearman's rho	—	
	p-value	—	
2. MAP	Pearson's r	0.167	—
	p-value	0.585	—
	Spearman's rho	0.129	—
	p-value	0.674	—

Assumption checks

Shapiro-Wilk Test for Bivariate Normality

			Shapiro-Wilk	p
iCarT	-	MAP	0.819	0.002

Correlation Table

Variable		iCarT	VC
1. iCarT	Pearson's r	—	
	p-value	—	
	Spearman's rho	—	
	p-value	—	
2. VC	Pearson's r	0.288	—
	p-value	0.341	—
	Spearman's rho	0.216	—
	p-value	0.479	—

Assumption checks

Shapiro-Wilk Test for Bivariate Normality

			Shapiro-Wilk	p
iCarT	-	VC	0.895	0.067

Correlation Table

Variable		iCarT	SVR
1. iCarT	Pearson's r	—	
	p-value	—	
	Spearman's rho	—	
	p-value	—	
2. SVR	Pearson's r	0.210	—
	p-value	0.491	—
	Spearman's rho	0.172	—
	p-value	0.573	—

Assumption checks

Shapiro-Wilk Test for Bivariate Normality

			Shapiro-Wilk	p
iCarT	-	SVR	0.891	0.055

Supplemental Figure 3. Individual and mean responses of stroke volume and cardiac output across incremental exercise stages

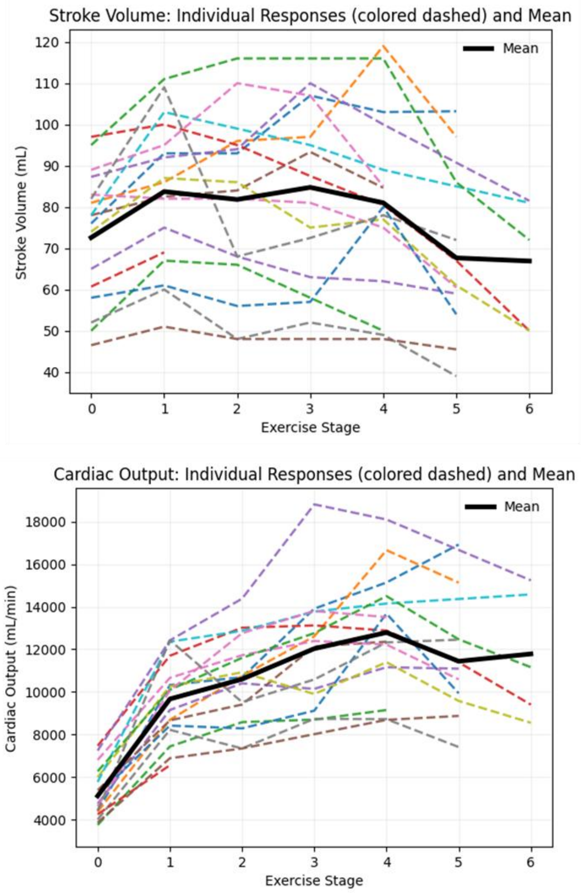

Supplement: Supplementary file 1 — Supplementary Figures S1–S3. [file EPH-9999-0-s001.pdf]
